# Placental growth factor testing for suspected pre‐eclampsia: a cost‐effectiveness analysis

**DOI:** 10.1111/1471-0528.15855

**Published:** 2019-07-17

**Authors:** KE Duhig, PT Seed, JE Myers, R Bahl, G Bambridge, S Barnfield, J Ficquet, JC Girling, A Khalil, AH Shennan, LC Chappell, RM Hunter

**Affiliations:** ^1^ Department of Women and Children's Health School of Life Course Sciences King's College London London UK; ^2^ The Division of Developmental Biology and Medicine University of Manchester Manchester UK; ^3^ University Hospitals Bristol NHS Foundation Trust Bristol UK; ^4^ Kingston Hospital NHS Foundation Trust Kingston upon Thames UK; ^5^ North Bristol NHS Trust Bristol UK; ^6^ Royal United Hospitals Bath Bath UK; ^7^ West Middlesex University Hospital Chelsea and Westminster Hospital NHS Foundation Trust London UK; ^8^ Fetal Medicine Unit St George's Hospital St George's University of London London UK; ^9^ Vascular Biology Research Centre Molecular and Clinical Sciences Research Institute St George's University of London London UK; ^10^ Research Department of Primary Care and Population Health University College London London UK

**Keywords:** Economic analysis, placental growth factor, pre‐eclampsia

## Abstract

**Objective:**

To calculate the cost‐effectiveness of implementing PlGF testing alongside a clinical management algorithm in maternity services in the UK, compared with current standard care.

**Design:**

Cost‐effectiveness analysis.

**Setting:**

Eleven maternity units participating in the PARROT stepped‐wedge cluster‐randomised controlled trial.

**Population:**

Women presenting with suspected pre‐eclampsia between 20^+0^ and 36^+6^ weeks’ gestation.

**Methods:**

Monte Carlo simulation utilising resource use data and maternal adverse outcomes.

**Main outcome measures:**

Cost per maternal adverse outcome prevented.

**Results:**

Clinical care with PlGF testing costs less than current standard practice and resulted in fewer maternal adverse outcomes. There is a total cost‐saving of UK£149 per patient tested, when including the cost of the test. This represents a potential cost‐saving of UK£2,891,196 each year across the NHS in England.

**Conclusions:**

Clinical care with PlGF testing is associated with the potential for cost‐savings per participant tested when compared with current practice via a reduction in outpatient attendances, and improves maternal outcomes. This economic analysis supports a role for implementation of PlGF testing in antenatal services for the assessment of women with suspected pre‐eclampsia.

**Tweetable abstract:**

Placental growth factor testing for suspected pre‐eclampsia is cost‐saving and improves maternal outcomes.

## Tweetable abstract

Placental growth factor testing for suspected pre‐eclampsia is cost‐saving and improves maternal outcomes.

## Introduction

Hypertension in pregnancy affects up to 10% of pregnant women. Pre‐eclampsia complicates 2.8% of singleton pregnancies^1^ and is associated with maternal and perinatal adverse outcomes.^2^ In 2012, the cost of pre‐eclampsia within the first 12 months of delivery was US$2.18 billion in the USA.^3^

Around 10% of pregnant women will be investigated for suspected pre‐eclampsia, making it among the most common clinical presentations to obstetric emergency services. The diagnosis of pre‐eclampsia is clinically challenging. Commonly used methods of investigation such as blood pressure measurement and proteinuria assessment demonstrate highly variable test performance.^4^, ^5^, ^6^ Ultrasound scanning and maternal admission are costly. Iatrogenic preterm birth associated with the disease is resource‐intensive.^7^, ^8^, ^9^ In prospective observational cohort studies, placental growth factor (PlGF) <5th centile has good test performance in assessing women with suspected pre‐eclampsia for determining need for delivery for pre‐eclampsia within 14 days of testing.^10^ We recently reported in the Placental growth factor for the Assessment of hypeRtensive pRegnant wOmen: a stepped wedge Trial (PARROT) that PlGF testing used alongside a clinical management algorithm reduced the time to diagnosis of pre‐eclampsia from 4.1 days (usual care) to 1.9 days (intervention).^11^ Where PlGF was implemented, severe maternal adverse outcomes^12^ were reduced from 5.4% (24/447; usual care group) to 3.8% (22/573; intervention group), with no evidence of a difference in gestation at delivery (36.6 versus 36.8 weeks).

National guidelines have approved PlGF testing to rule out suspected pre‐eclampsia in the UK.^13^ Hypothetical economic models have found that PlGF‐based testing gives a cost‐saving of between £330 and £1032^13^, ^14^, ^15^ per woman tested.^16^, ^17^, ^18^, ^19^, ^20^, ^21^, ^22^, ^23^ These models have not examined trial data where use of PlGF has been incorporated into clinical care.

The PARROT trial provides an opportunity to assess the cost implications of implementing PlGF testing into the health service with clinical data, and the benefit of a controlled comparison group. The aim of this study was to conduct a within‐trial analysis to calculate the incremental cost per maternal adverse event prevented associated with implementing PlGF testing in maternity services in the National Health Service (NHS) in England, compared with current standard care.

The use of PlGF testing alongside a clinical management algorithm may enable clinicians to target resources to those at greatest need (women testing with a PlGF <100 pg/ml). This could aid clinical decision making, enabling appropriate risk stratification of care. We anticipated that women with normal PlGF (>100 pg/ml) could be managed with less intensive surveillance, providing a cost‐saving per woman tested.^13^

## Methods

This economic evaluation is a within‐trial analysis using data from the PARROT trial and is from an NHS cost perspective.

### Trial processes

The PARROT study was a multicentre stepped‐wedge cluster‐randomised controlled trial of PlGF testing alongside a clinical management algorithm in the assessment of women with suspected pre‐eclampsia. Eleven UK maternity units (size 3000–9000 deliveries per year) took part. Women aged 18 years or older were invited to participate in the trial if they presented to antenatal assessment services with suspected pre‐eclampsia (for example to maternity triage or the acute obstetric assessment unit) with a live, singleton fetus between 20^+0^ weeks and 36 weeks^+6^ of gestation. Women with a diagnosis of pre‐eclampsia at the time of clinical presentation were excluded. The maternity units (clusters) were randomly allocated to the order in which the intervention (revealed PlGF alongside a clinical management algorithm) was introduced. At the beginning of the trial, all units followed ‘usual care’, and every 6 weeks, a randomly allocated cluster would begin adoption of revealed PlGF testing. By the end of the trial, all clusters were recruiting with revealed PlGF testing. The PARROT trial reported outcomes in over 99% of the participants enrolled. The trial was approved by the London South East Research Ethics Committee (ref. 15/LO/2058).

### The comparator group and the intervention

Within ‘usual care’, an additional blood sample was collected at enrolment and was processed on an electronically masked Triage (Alere, San Diego, CA, USA; now Quidel Cardiovascular Inc., San Diego, CA, USA) instrument according to the manufacturer's instructions, so that the result was recorded but not revealed to the clinical team. Women were managed by an attending obstetrician following local hospital practice based on the NICE guidance on the Management of Hypertension in Pregnancy.^24^

Under the intervention conditions (PlGF testing alongside a management algorithm) women provided a blood sample which was processed within 4 hours on an unmasked Triage instrument (Alere, now Quidel Cardiovascular Inc.) according to the manufacturer's instructions. A result was given to the attending obstetrician to inform clinical care. The trial was deliberately pragmatic to reflect how PlGF may be adopted within a healthcare service. No prescriptive care schedules were recommended following PlGF testing. The management algorithm provided simple guidance only, incorporating serum PlGF concentrations categorised as normal (>100 pg/ml), low (12–100 pg/ml) or very low (<12 pg/ml) into the national (NICE) guidance for the Management of Hypertension in Pregnancy (Figure S1). Women with a serum PlGF concentration of >100 pg/ml followed a care pathway involving outpatient management and routine surveillance unless clinical parameters such as severe hypertension indicated otherwise. Those with low PlGF concentrations were advised to ‘increase surveillance’ with a greater frequency of antenatal care visits and fetal ultrasound scanning. Those with very low PlGF were ‘assessed as pre‐eclampsia’, which included consideration for admission, intensive monitoring, and fetal ultrasound scanning.

The primary outcome in the trial was the time, in days, from presentation with suspected pre‐eclampsia to the woman  receiving a documented diagnosis of pre‐eclampsia in the clinical notes. Secondary outcomes included adverse maternal and perinatal outcomes. Participants were followed up from the point of recruitment to the primary postnatal discharge of the woman and infant pair. All data were taken from clinical records including handheld notes, electronic maternity records, and neonatal records.

### Resource use and costs

Resource use data was prespecified. Maternal resource use included maternity outpatient appointments, antenatal hospital admission, and hospital admission associated with delivery. This included both standard and intensive care admissions. Infant resource use included routine care, and admission to a neonatal unit (special care, high‐dependency, and intensive care).^25^ Information was collected on mode of delivery based on reference cost categories (spontaneous vaginal, assisted delivery, planned caesarean, and emergency caesarean).^26^, ^27^ The cost of delivery has been reported separately as it is included in the NHS Reference Costs used to calculate the cost per bed day, and hence it would be double‐counting to include this in the Monte Carlo simulation.

Unit costs were obtained from NHS Reference Costs 2016/2017^27^ and are reported in Table S1. All costs are weighted, based on the sum of the unit costs for each relevant Healthcare Resource Group multiplied by activity. The sum of costs is then divided by the sum of the activity to give a weighted cost per patient based on the frequency of each Health Care Resource Group. All neonatal admissions were given the same cost per bed day, as a weighted cost per bed day derived from the relevant Health Care Resource Group multiplied by length of stay provides a closer estimate of total cost then using activity costs based on ward type.^27^

The cost of the PlGF test was estimated at £70 per test 2017/2018 (all prices given in Pound Sterling [£] [Quidel, Cardiovascular Inc.]). We conducted sensitivity analyses varying the cost of the test between UK£50 and £200.

### Effectiveness outcome

Core outcome sets and patient involvement were not relevant to this secondary economic analysis. Quality‐adjusted life years (QALYs) are considered the gold standard effectiveness outcome in economic evaluations.^28^ The use of QALYs in economic evaluations of interventions in maternity services presents a number of challenges.^29^ Patient‐reported outcomes were also not collected as part of this trial, so QALYs calculated using a preference‐based measure of health‐related quality of life was not possible. Instead, effectiveness has been calculated as the mean number of maternal adverse events avoided per 1000 women with the intervention compared with current practice. This was calculated based on the maternal adverse events reported in the PARROT Trial.^11^

### Statistical analysis

Cost‐savings associated with PlGF testing for suspected pre‐eclampsia may differ by PlGF sub‐group (normal, low, and very low), as demonstrated in previous economic models.^19^ Cost‐savings may also differ based on final diagnosis, e.g. pre‐eclampsia; gestational hypertension/chronic hypertension/small for gestational age or none of the above.^19^ Each participant was allocated to one of the nine subgroups groups based on serum PlGF concentration and final diagnosis. These were included as covariates in each analysis.

Healthcare resource use was analysed using generalised linear mixed models (GLMM). Each of the nine categories was analysed separately to determine the most appropriate form of general linear model based on the Akaike information criterion (AIC). For all models except one, the two‐part negative binomial model was the most appropriate. This model accounts for the probability that a participant uses a service, using a count (Poisson) model for values greater than zero. For neonate intensive care and high dependency unit (ICU/HDU) admissions, the complex GLMM would not converge. As a result this was modelled using logistic regression, and linear regression was used for length of stay for admitted infants only.

To account for the stepped‐wedge cluster‐randomised nature of the trial, all models included fixed effect of linear time and a random effect for centre.^30^ Mean adjusted resource use (with standard errors) were calculated for each of the nine groups, and by trial allocation. The mean cost of delivery and 95% confidence intervals by trial allocation were calculated using a GLMM with log link and family gamma. All statistical analyses were undertaken using STATA version 14.2 (StataCorp, College Station, TX, USA)

### Probabilistic model

A simple costing analysis of the data does not allow for exploration of the complex inter‐relationship between PlGF concentration, participant diagnosis, and resource use. Due to the skewed, over‐dispersed nature of the resource use data, and the fact that these analyses were not the primary powered end point in the PARROT trial, it is unlikely that we would find statistically significant results, and any significant results would require circumspect interpretation.^31^ Instead we have used predicted adjusted means (and standard errors) and Monte Carlo simulation to calculate the probability that PlGF testing is cost‐saving compared with current practice for maternal costs, infant costs; delivery costs, and all costs combined. Values used in the model and their distributions are reported in Tables S2–S8.

Total cost differences between trial arms were calculated as total costs and as weighted costs. Weighting accounted for the proportion of participants in each of the nine subgroups in each arm of the trial.

The number of adverse events was calculated using a beta distribution with Monte Carlo simulation for 5000 iterations of the model. The probability that PlGF testing was cost‐effective compared with current practice was calculated as willingness to pay per adverse event avoided, multiplied by the number of adverse events avoided, minus the total cost per 1000 women with suspected pre‐eclampsia. The probability that PlGF testing is cost‐effective includes the cost of the PlGF test.

The model was run 5000 times, which was used to generate the cost‐effectiveness acceptability curve (the probability that PlGF testing is cost‐effective for a range of values of willingness to pay for an adverse event avoided).

The model was developed and run in Microsoft EXCEL FOR OFFICE 365 (Redmond, WA, USA).

## Results

In all, 1005 women were included in the analysis, 434 with usual care and 571 with clinical care with PlGF testing. Among all participants, 236 (23.5%) had a PlGF <12 pg/ml, 385 (38.3%) a PlGF 12–100 pg/ml, and 384 (38.2%) had a PlGF > 100 pg/ml. There was no contamination between trial arms (i.e. no duplication of National Health Service numbers in the trial database).

### Participant demographics

**Figure 1:**
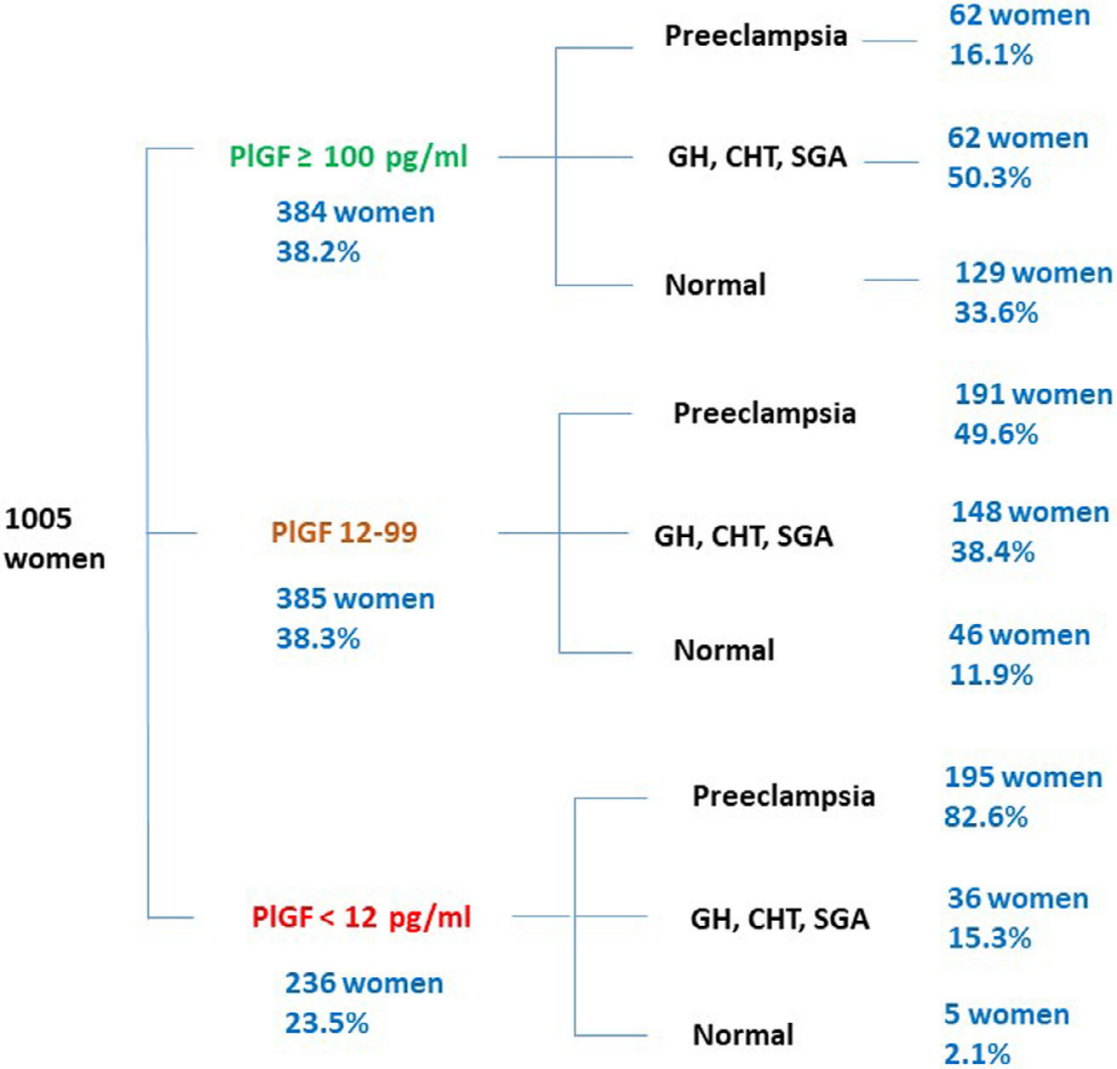
Proportion of women in each diagnostic and PlGF category in the PARROT trial. CHT, chronic hypertension; GH, gestational hypertension; SGA, small for gestational age.

Women had a mean age of 31.49 years (SD 5.98 years) and a mean gestational age of 32–33 weeks’ gestation at trial entry. In all, 66% of the women were white, 14% were black, 12% were Asian (Indian, Pakistani, Sri Lankan or Bangladeshi), 2% were of mixed ethnicity, and 6% were from Chinese or other ethnic backgrounds. The median body mass index at booking was 28.4 kg/m^2^ (IQR 24.2–34.1). There was a history of pre‐eclampsia in a previous pregnancy in 39%. Full demographics and outcomes have been reported in the main trial paper.^11^ The proportion of women in each diagnostic and PlGF subcategory in the trial are reported in Figure 1.

### Costs

Descriptive statistics and average costs by diagnosis, PlGF test result, and trial arm allocation compared with current practice for appointments, length of stay or probability of admission are reported in Tables S2–S4. Cost differences (actual and weighted) and the proportion of times clinical care with PlGF testing costs less than current practice is reported in Tables 1 and 2. There was no difference in the cost of delivery between the two groups, with an average cost of delivery of £3,372 with PlGF testing, (95% CI £3,258 to £3,484) and an average cost of £3,318 with usual care (95% CI £3,187 to £3,450).

**Table 1:** Results of model and Monte Carlo Simulation: total cost difference per patient for PlGF minus current practice (actual, weighted, and percentage of 5000 iterations that are cost‐saving) for maternal costs. All prices given in GBP (£)

	PlGF (pg/ml)	PlGF >100			PlGF 12–100			PlGF < 12			Total
Resource use	Statistic	Normal	GH/CHT/SGA	Pre‐eclampsia	Normal	GH/CHT/SGA	Pre‐eclampsia	Normal	GH/CHT/SGA	Pre‐eclampsia	
Outpatient	Actual	−£647	−£479	−£335	−£608	−£139	−£590	−£638	−£282	−£345	−£4,062
	Weighted	−£82	−£93	−£21	−£28	−£20	−£113	−£3	−£10	−£67	−£436
	Percentage <0	99.9%	98.4%	86.1%	98.2%	76.3%	100%	100%	84.3%	99.7%	100%
Antenatal ward	Actual	£112	−£966	£1,422	−£174	£13	£777	−£770	£3,341	£582	£4,336
	Weighted	£14	−£187	£88	−£8	£2	£148	−£4	£119	£113	£285
	Percentage <0	32.0%	99.3%	11.7%	67.2%	49.4%	18.8%	100%	0.6%	28.1%	17.3%
Labour ward	Actual	£106	£107	£119	£275	£45	£13	−£166	−£54	−£58	£388
	Weighted	£13	£21	£7	£13	£7	£3	−£1	−£2	−£11	£49
	Percentage <0	16.3%	18.8%	26.1%	4.9%	37.3%	46.8%	100%	62.8%	68.4%	17.4%
Maternal postnatal	Actual	−£116	−£464	£551	−£251	£3	£361	−£1,276	−£575	£418	−£1,350
	Weighted	−£15	−£90	£34	−£11	£0	£69	−£6	−£21	£81	£41
	Percentage <0	71%	98.6%	9.1%	74%	50.2%	9.8%	100%	80.6%	10.2%	36%
Maternal ICU/HDU	Actual	£43	−£84	−£77	−£385	£52	−£336	−£0	£211	−£20	−£596
	Weighted	£5	−£16	−£5	−£18	£8	−£64	−£0	£8	−£4	−£86
	Percentage <0	20.2%	78.6%	59.5%	94.1%	20.9%	89.4%	0%	0%	51.2%	78.7%
Total Maternal	Actual	−£502	−£1,887	£1,679	−£1,142	−£26	£225	−£2,850	£2,641	£577	−£1,284
	Weighted	−£64	−£365	£103	−£52	−£4	£43	−£14	£94	£112	−£147
	Percentage <0	88.7%	100%	10.3%	95.1%	52.4%	40.9%	100%	5.4%	30.9%	66.6%

Actual values derived from adjusted models accounting for PlGF and final diagnosis. CHT, chronic hypertension; GH, gestational hypertension; SGA, small for gestational age; Weighted, weighted for proportion of women in each of the nine PlGF/Diagnostic groups—to calculate the weighted average cost per woman.

**Table 2:** Results of model and Monte Carlo simulation: total cost difference per patient for PlGF minus current practice (actual, weighted, and percentage of 5000 iterations that are cost‐saving) for infant and total costs. All prices given in GBP (£)

	PlGF (pg/ml)	PlGF >100			PlGF 12–100			PlGF < 12			Total
Resource use	Statistic	Normal	GH/CHT/SGA	Pre‐eclampsia	Normal	GH/CHT/SGA	Pre‐eclampsia	Normal	GH/CHT/SGA	Pre‐eclampsia	
Infant ICU/HDU	Actual	£187	−£976	£84	−£86	−£941	£1,061	£0	−£7,079	£818	−£6,932
	Weighted	£24	−£189	£5	−£4	−£138	£202	£0	−£253	£158	−£195
	Percentage <0	0%	97%	23%	100%	95.6%	0.6%	0%	95.2%	16.5%	65%
Infant SCBU	Actual	−£183	−£105	£393	−£379	−£376	£558	−£0	−£292	£610	£227
	Weighted	−£23	−£20	£24	−£17	−£55	£106	−£0	−£10	£118	£122
	Percentage <0	98.1%	74.6%	15.1%	88.6%	76.5%	21.6%	0%	53.1%	35.1%	37.7%
Total Infant	Actual	£4	−£1,080	£477	−£465	−£1,317	£1,619	−£0	−£7,371	£1,428	−£6,705
	Weighted	£0	−£209	£29	−£21	−£194	£309	−£0	−£264	£276	−£72
	Percentage <0	70.6%	97.8%	13.8%	91.7%	94.9%	2.9%	0%	89.3%	22.7%	50.4%
Total maternal & infant costs	Actual	−£498	−£2967	£2157	−£1607	−£1343	£1845	−£2850	−£4730	£2005	−£7990
	Weighted	−£63	−£575	£133	£73	−£197	£352	−£14	−£169	£388	−£219
	Percentage <0	89.0%	100%	13.2%	97.9%	88.2%	8.0%	100%	75.5%	18.6%	59.9%

Actual values derived from adjusted models accounting for PlGF and final diagnosis. CHT, chronic hypertension; GH, gestational hypertension; SGA, small for gestational age; Weighted, weighted for proportion of women in each of the nine PlGF/Diagnostic groups—to calculate the weighted average cost per woman.

Outpatient appointments had lower costs for all subgroups, with 100% of iterations of the model being cost‐saving with PlGF testing. Maternal inpatient admission costs were greater with PlGF testing. This was mostly due to increased costs for women with a final pre‐eclampsia diagnosis from appropriate management. Overall there was a reduction in costs for women admitted to intensive or high‐dependency care (£86 weighted cost saving per woman, in 78.74% of iterations) and infant neonatal admissions, although with lower certainty (£71 weighted cost saving per infant in 50.4% of iterations).

Overall, the average weighted cost‐saving per woman with PlGF testing was £147 in 66.6% of iterations. For women and infants combined, the average weighted cost‐saving with PlGF testing was £149 per woman in 55.5% of iterations of the model, when accounting for a PlGF test cost of £70. Without accounting for the cost of PlGF testing, the average weighted cost saving per woman tested was £219 in 59.9% of iterations. Table 3 presents the probability that PlGF testing is cost‐saving for a range of PlGF testing prices.

**Table 3:** Probability that PlGF plus management algorithm is cost‐saving for a range of costs per PlGF test. All prices given in GBP (£)

Cost per PlGF test	Probability cost‐saving—Maternal costs only	Probability cost‐saving—Infant costs only	Probability cost‐saving—Maternal and Infant costs
£50	60.7%	46.6%	56.9%
£100	55.3%	42.2%	53.5%
£150	49.5%	38.2%	50.0%
£200	43.6%	34.4%	46.7%

### Cost‐effectiveness

Clinical care with PlGF testing resulted in an average of 15 fewer maternal adverse events per 1000 women tested compared with usual care. PlGF testing dominated usual care in that it cost less and resulted in fewer maternal adverse events. There is a 72% probability that the intervention is cost‐effective at a £20,000 willingness to pay for an adverse event prevented (see Figure S2).

## Discussion

### Main findings

The use of PlGF testing for suspected preterm pre‐eclampsia has a 59.9% probability of representing a cost‐saving compared with current practice, with a total cost‐saving of £149 per woman when including the cost of the test (in this instance, Triage PlGF at £70 per test). Given that there were 646 794 births in England in 2017,^32^ 10% of pregnant women have suspected pre‐eclampsia, and 30% of these present before 37 weeks’ gestation, PlGF testing could be performed in approximately 38 800 pregnant women per year. This would result in a potential cost saving of £2,891,196 each year across the English NHS. The majority of cost‐savings associated with PlGF testing are through a reduction seen in maternal outpatient appointments in women testing with a PlGF >100 pg/ml. Our resource use data suggest that where PlGF testing is implemented, high‐risk women (for example those with low or very low PlGF) are more appropriately managed, as shown by the increased antenatal inpatient costs in these groups. With different commercial assay prices, the magnitude of the cost savings will depend on the cost per test. PlGF testing results in fewer maternal adverse events for a lower cost than usual care, i.e. PlGF testing dominates usual care, and hence no incremental cost per adverse event prevented is reported.

### Strengths and limitations

The strengths of this study are in the direct comparison of resource use between women undergoing PlGF testing against women with usual care using trial data. The trial included NHS maternity units participating from across the UK, with prospective recruitment of an ethnically and socio‐demographically varied group of participants, enabling generalisability to the broader NHS setting.

Our analysis evaluates the impact that clinical decisions have on resource use following PlGF testing as compared with usual care. We report the cost‐savings and maternal adverse events averted. Usually a cost per adverse event reported should be avoided due to double‐counting: adverse events are included in both the costs and the effectiveness estimate. However, within our trial we did not include resource use assessment beyond the primary postnatal discharge of the mother and infant pair. We therefore did not capture any ongoing costs associated with maternal adverse events. In the PARROT trial there were five serious adverse events with usual care (two eclamptic fits, two strokes, and one cardiac arrest in four women) compared with no such corresponding events with PlGF testing. It was not possible to include community rehabilitation costs or resource use associated with ongoing medical management and readmissions. We were unable to assess health‐related quality of life and wellbeing scores, and any impact on loss of earnings associated with these serious adverse events, which may be significant. Our estimated cost‐saving is therefore likely to be a conservative estimate.

### Interpretation

In this study, we have shown a modest cost‐saving in comparison with other studies.^16^, ^17^, ^19^, ^20^, ^21^, ^22^, ^23^ Previous health economic studies of the implementation of PlGF testing in clinical care were based on hypothetical assumptions of reduction in resource use, with greater cost‐savings than we have presented in this analysis. Current ‘usual care’ relies upon imperfect stratification of pregnancies with suspected pre‐eclampsia by obstetricians causing resource‐intensive investigation of women. PlGF testing enables more appropriate stratification, targeting resource use to where it is more clinically appropriate. The conservative cost‐saving presented in our analysis is due to a redistribution in maternity and neonatal resource use with PlGF testing, rather than an overall reduction in resource use that was anticipated by hypothetical models.

The PARROT trial evaluated PlGF testing in women presenting with suspected pre‐eclampsia. The PARROT trial did not assess indications for or frequency of repeat PlGF testing. There is currently no mandate for repeated PlGF testing outside of research settings but it may impact further on maternal and perinatal outcomes, and subsequently on health resource use. The optimum frequency of repeated PlGF measurement remains uncertain but is likely that repeat sampling will impact on costs associated with the implementation of PlGF testing. This could be both in the cost of additional tests and in cost‐savings associated with better stratification of care and avoidance of further morbidity.

## Conclusion

Clinical care with PlGF testing is cost‐saving, and is associated with reduced numbers of maternal adverse events compared with usual care. Given that PlGF testing has also been shown to be associated with an improvement in the time to diagnosis of pre‐eclampsia,^11^ this analysis supports a role for implementation of PlGF in antenatal services for the assessment of women with suspected pre‐eclampsia.

### Disclosure of interests

KED, LCC, PS, JEM, JCG, RB, GB, SB, JF, AK, and AS have no conflicts of interest. RMH has received consulting fees from Alere, outside the submitted work. Completed disclosure of interests forms are available to view online as Supporting Information.

### Contribution to authorship

All authors were involved in the study conception and in securing funding for the study. Clinical study data analysis was undertaken by KED, LCC, and PS. Health economic analysis was undertaken by RMH. The manuscript was written by KED and RMH, with assistance from LCC, JCG, JEM, PS, GB, SB, RB, JF, AK, and AHS. All authors approved the final version of the manuscript.

### Details of ethics approval

The trial was approved by the London South East Research Ethics Committee (ref. 15/LO/2058) on 21 January 2016.

### Funding

This study was supported by grants from the National Institute for Health Research, Research for Patient Benefit Programme (PB‐PG‐0214‐33054) and National Institute for Health Research Professorship (Chappell RP‐2014‐05‐019). The views expressed in this publication are those of the author(s) and not necessarily those of the NHS, the National Institute for Health Research or the Department of Health.

### Acknowledgements

We thank all women who participated in the PARROT Trial.

## Supporting information


**Figure S1.** PARROT Trial Clinical Management Algorithm.

**Figure S2.** Probability that PlGF testing in women presenting with pre‐eclampsia compared with current practice is cost‐effective for a range of values of willingness to pay for a maternal adverse event prevented.

**Table S1.** Unit costs (per day for inpatient stays) for resource use taken from 2016/2017 National Schedule of Reference Costs (2016/2017 GBP).
**Table S2.** Model inputs for revealed PlGF testing for antenatal costs.
**Table S3.** Model inputs for revealed PlGF testing for postnatal costs.
**Table S4.** Model inputs for revealed PlGF testing for infant costs.
**Table S5.** Model inputs in concealed PlGF testing for antenatal costs.
**Table S6.** Model inputs in concealed PlGF testing for postnatal costs.
**Table S7.** Model inputs in concealed PlGF testing for infant costs.
**Table S8.** Results of model: actual total cost per patient for PlGF and usual care.
