# Supplementary figures and images for: Placental growth factor testing for suspected pre‐eclampsia: a cost‐effectiveness analysis
Source: BJOG. 2019 Jul 17;126(11):1390–8. doi: 10.1111/1471-0528.15855 (PMC6771855; doi:10.1111/1471-0528.15855)

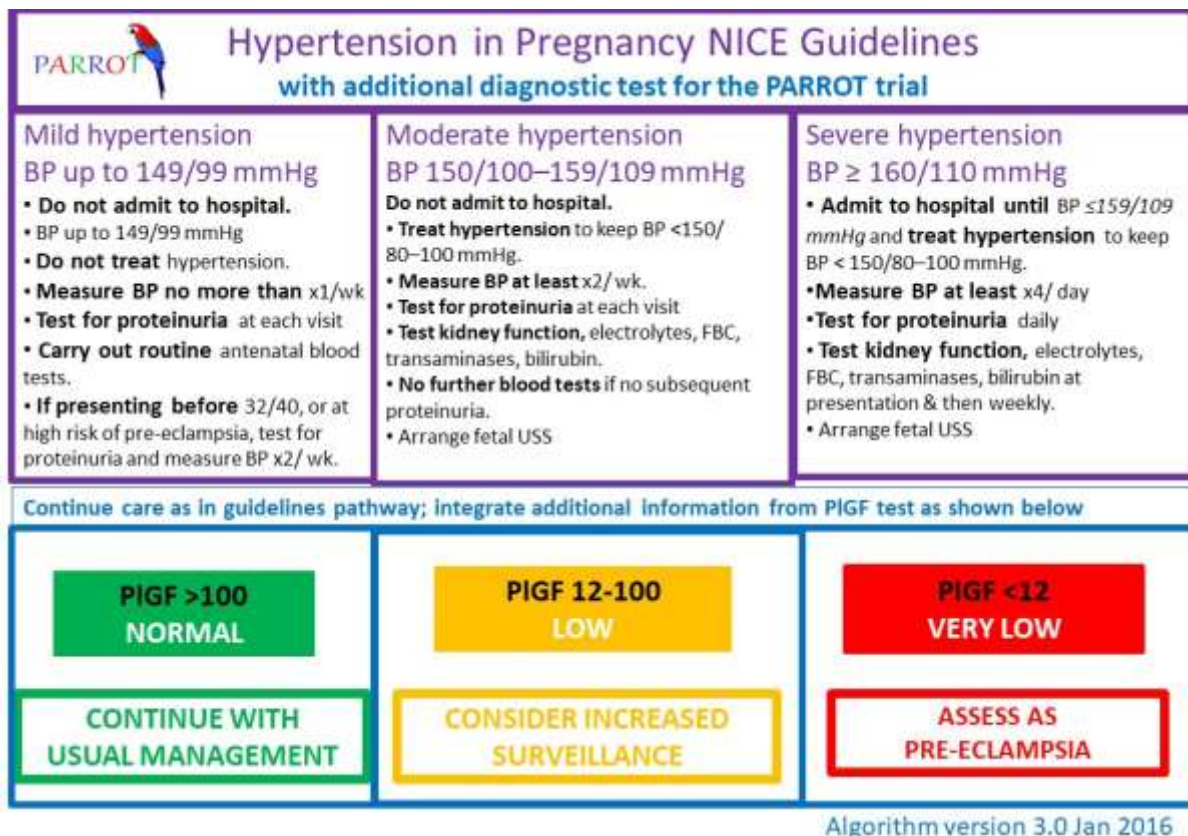

**Figure S1.** PARROT Trial Clinical Management Algorithm

Supplement: Supplementary file 1 — Figure S1. PARROT Trial Clinical Management Algorithm. [file BJO-126-1390-s001.pdf]
