# Supplementary material for: Placental growth factor testing for suspected pre‐eclampsia: a cost‐effectiveness analysis
Source: BJOG. 2019 Jul 17;126(11):1390–8. doi: 10.1111/1471-0528.15855 (PMC6771855; doi:10.1111/1471-0528.15855)
Supplement: Supplementary file 2 — Figure S2. Probability that PlGF testing in women presenting with pre‐eclampsia compared with current practice is cost‐effective for a range of values of willingness to pay for a maternal adverse event prevented. [file BJO-126-1390-s002.pdf]

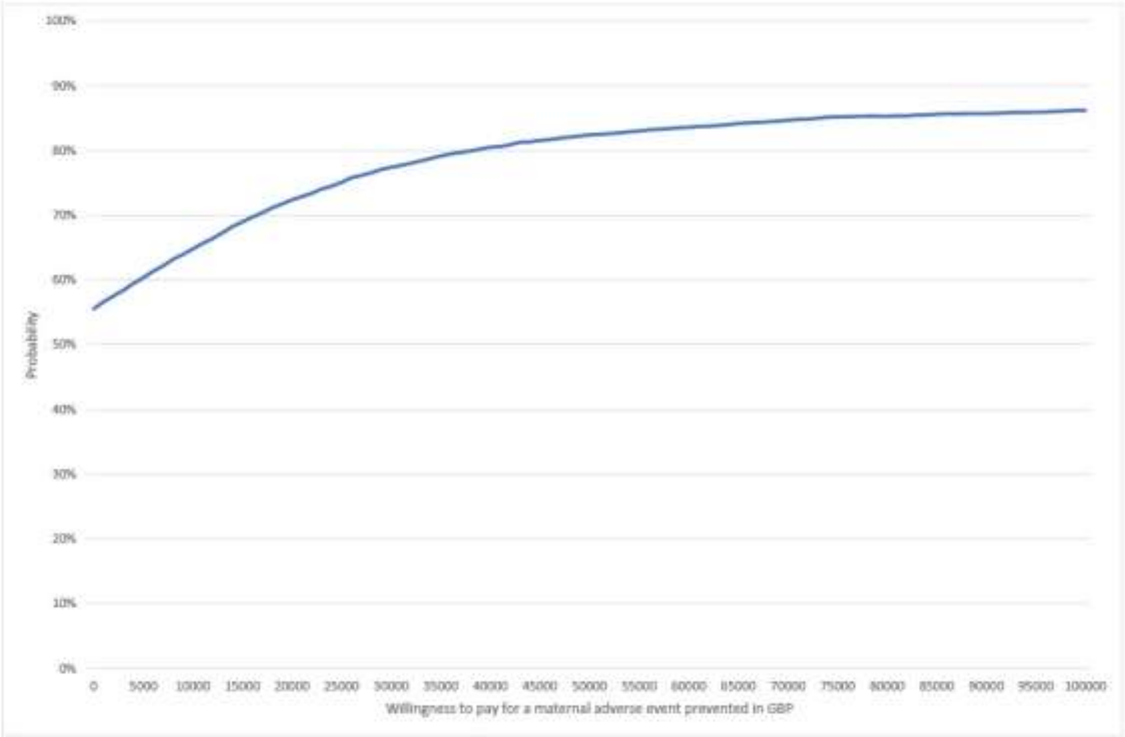

Figure S2. Probability that PlGF testing in women presenting with preeclampsia compared to current practice is cost-effective for a range of values of willingness to pay for a maternal adverse event prevented.
