# Supplementary material for: Placental growth factor testing for suspected pre‐eclampsia: a cost‐effectiveness analysis
Source: BJOG. 2019 Jul 17;126(11):1390–8. doi: 10.1111/1471-0528.15855 (PMC6771855; doi:10.1111/1471-0528.15855)
Supplement: Supplementary file 3 — Table S1. Unit costs (per day for inpatient stays) for resource use taken from 2016/2017 National Schedule of Reference Costs (2016/2017 GBP). Table S2. Model inputs for revealed PlGF testing for antenatal costs. Table S3. Model inputs for revealed PlGF testing for postnatal costs. Table S4. Model inputs for revealed PlGF testing for infant costs. Table S5. Model inputs in concealed PlGF testing for antenatal costs. Table S6. Model inputs in concealed PlGF testing for postnatal costs. Table S7. Model inputs in concealed PlGF testing for infant costs. Table S8. Results of model: actual total cost per patient for PlGF and usual care. [file BJO-126-1390-s003.pdf]

Table S1. Unit costs (per day for inpatient stays) for resource use taken from 2016/2017 National Schedule of Reference Costs (2016/2017 GBP)[26]

Resource	Unit cost (£)
Outpatient	129
Antenatal ward	809
Labour Ward	724
Maternal Intensive Care/High Dependency Care	1012
Neonatal Unit	340

Table S2. Model inputs for revealed PlGF testing for antenatal costs

	Diagnosis	Outpatient visits, n mean	Outpatient visits, n SE	Cost	Antenatal Ward	Antenatal ward se	Cost	Labour ward	Labour ward SE	Cost
PlGF>100	Normal	6.039471	0.821633	778.0047	1.087079	0.213071	879.4469	0.538285	0.115798	389.7185
	GH/CHT/SGA	7.986798	0.945846	1028.859	1.542404	0.235514	1247.805	0.692995	0.123705	501.7284
	Pre-eclampsia	8.376477	1.485062	1079.058	5.153079	1.179306	4168.841	0.794508	0.196133	575.2239
Plgf<100>12	Normal	5.36796	1.098899	691.5006	0.81746	0.274417	661.3251	0.704587	0.2004	510.1206
	GH/CHT/SGA	7.483879	0.990128	964.0733	2.372329	0.412841	1919.214	0.671975	0.132991	486.5096
	Pre-eclampsia	5.180283	0.622814	667.3241	5.709159	0.813716	4618.71	0.782674	0.140804	566.6557
PlGF<12	Normal									
	GH/CHT/SGA	5.698915	1.349894	734.1342	5.921327	1.875136	4790.354	0.422098	0.165004	305.5992
	Pre-eclampsia	4.425083	0.539258	570.0392	6.322338	0.906354	5114.771	0.577905	0.108955	418.4029

Table S3. Model inputs for revealed PlGF testing for postnatal costs

	**Diagnosis**	**Postnatal ward Mean**	**Postnatal ward SE**	**Cost**	**High need**	**High need SE**	**Cost**
PlGF>100	Normal	1.528936	0.176884	1106.95	0.097257	0.041836	98.42439
	GH/CHT/SGA	1.840509	0.16592	1332.529	0.175934	0.051856	178.0448
	Pre-eclampsia	3.165373	0.436271	2291.73	0.528301	0.204471	534.6404
Plgf<100>12	Normal	1.932686	0.341121	1399.265	0.152544	0.09401	154.3747
	GH/CHT/SGA	2.826986	0.275142	2046.738	0.134063	0.051758	135.672
	Pre-eclampsia	3.387897	0.273765	2452.837	0.521421	0.131584	527.6775
PlGF<12	Normal						
	GH/CHT/SGA	3.221175	0.585443	2332.131	0.208702	0.136497	211.2065
	Pre-eclampsia	4.201791	0.321327	3042.097	1.214391	0.289255	1228.964

Table S4. Model inputs for revealed PlGF testing for infant costs

	Diagnosis	Infant admitted HDU/ICU	Infant admit SE	Infant los HDU/ICU	Infant LOS SE	Infant ICU/HDU cost	Infant los SCBU	Infant LOS SE	Infant SCBU cost
PlGF>100	Normal	0.042349	0.024211	13	10.70074	187.1817	0.168241	0.075318	57.20197
	GH/CHT/SGA	0.073257	0.024801	8.666667	1.92713	215.8625	0.805477	0.237718	273.8621
	Pre-eclampsia	0.151931	0.063425	5.2	1.567997	268.6135	2.176834	1.07045	740.1236
Plgf<100>12	Normal	0	0	0	0	0	0.442301	0.291395	150.3825
	GH/CHT/SGA	0.116305	0.037999	7	1.744341	276.8054	2.375839	0.811442	807.7853
	Pre-eclampsia	0.292303	0.048112	14.39394	3.327992	1430.512	5.90239	1.638849	2006.813
PlGF<12	Normal								
	GH/CHT/SGA	0.408132	0.120391	19.57143	8.580245	2715.826	10.5525	6.652056	3587.85
	Pre-eclampsia	0.487437	0.053973	18.55556	3.177951	3075.189	13.17782	3.665785	4480.459

Table S5. Model inputs in concealed PlGF testing for antenatal costs

	Diagnosis	Outpatient visits, n mean	Outpatient visits, n SE	Cost	Antenatal Ward	Antenatal ward se	Cost	Labour ward	Labour ward SE	Cost
PlGF>100	Normal	11.06014	1.629282	1424.767	0.948858	0.215468	767.6262	0.392139	0.105223	283.9087
	GH/CHT/SGA	11.70156	1.521237	1507.395	2.736931	0.474298	2214.177	0.545693	0.114187	395.0815
	Pre-eclampsia	10.97693	2.015226	1414.048	3.395794	0.84151	2747.197	0.629727	0.170935	455.9221
Plgf<100>12	Normal	10.08389	2.167276	1299.007	1.03271	0.354972	835.4624	0.324179	0.133194	234.7055
	GH/CHT/SGA	8.561043	1.143293	1102.834	2.356203	0.423323	1906.168	0.609826	0.127806	441.514
	Pre-eclampsia	9.763741	1.214002	1257.765	4.748596	0.742103	3841.614	0.764099	0.144472	553.2074
PlGF<12	Normal	4.952535	2.149283	637.9856	0.952351	0.690364	770.4521	0.229167	0.235714	165.9172
	GH/CHT/SGA	7.88944	1.813436	1016.318	1.791595	0.607602	1449.4	0.496536	0.186018	359.492
	Pre-eclampsia	7.104001	0.909941	915.1374	5.602629	0.890069	4532.527	0.657822	0.128406	476.2629

Table S6. Model inputs in concealed PlGF testing for postnatal costs

	Diagnosis	Postnatal ward Mean	Postnatal ward SE	Cost	High need	High need SE	Cost
PlGF>100	Normal	1.689389	0.219839	1223.118	0.054563	0.035162	55.21776
	GH/CHT/SGA	2.481695	0.252854	1796.747	0.259343	0.089599	262.4553
	Pre-eclampsia	2.40467	0.370908	1740.981	0.60471	0.254159	611.9664
Plgf<100>12	Normal	2.279245	0.425869	1650.173	0.533331	0.28021	539.7306
	GH/CHT/SGA	2.823447	0.283944	2044.176	0.082609	0.04041	83.6
	Pre-eclampsia	2.889429	0.267679	2091.947	0.853006	0.237684	863.2418
PlGF<12	Normal	1.762265	0.708562	1275.88	3.44E-10	9.82E-06	3.48E-07
	GH/CHT/SGA	4.015174	0.682349	2906.986	3.29E-10	4.77E-06	3.33E-07
	Pre-eclampsia	3.624549	0.321932	2624.173	1.234034	0.329472	1248.842

Table S7. Model inputs in concealed PlGF testing for infant costs

	Diagnosis	Infant admitted HDU/ICU	Infant admit SE	Infant los HDU/ICU	Infant LOS SE	Infant ICU/HDU cost	Infant los SCBU	Infant LOS SE	Infant SCBU cost
PlGF>100	Normal	0	0	0	0	0	0.707839	0.314946	240.6653
	GH/CHT/SGA	0.097723	0.036142	35.85714	18.21511	1191.385	1.113436	0.391043	378.5682
	Pre-eclampsia	0.101768	0.055922	5.333333	5.876411	184.54	1.019797	0.561244	346.731
Plgf<100>12	Normal	0.101595	0.068008	2.5	1.535764	86.35592	1.555728	1.020697	528.9475
	GH/CHT/SGA	0.08774	0.035103	40.83333	15.7764	1218.125	3.481389	1.20688	1183.672
	Pre-eclampsia	0.16184	0.041748	6.714286	3.349861	369.4576	4.259938	1.329539	1448.379
PlGF<12	Normal	0	0	0	0	0	5.66E-09	3.43E-05	1.92E-06
	GH/CHT/SGA	0.398552	0.117149	72.28571	26.29157	9795.257	11.41148	7.374402	3879.903
	Pre-eclampsia	0.379988	0.058471	17.46875	3.781358	2256.889	11.38512	3.55963	3870.941

Table S8. Results of model: actual total cost per patient for PlGF and usual care

		Revealed PlGF				Usual Care			
	**Diagnosis**	**Total Mat**	**Weighted**	**Total inf**	**Weighted**	**Total Mat**	**Weighted**	**Total inf**	**Weighted**
PlGF>100	Normal	3252.544	413.4316	244.3837	31.06366	3754.638	477.2528	240.6653	30.59102
	GH/CHT/SGA	4288.966	830.5346	489.7246	94.83247	6175.856	1195.921	1569.954	304.0129
	Pre-eclampsia	8649.493	532.5408	1008.737	62.10695	6970.115	429.1431	531.271	32.70983
Plgf<100>12	Normal	3416.586	156.0704	150.3825	6.869507	4559.078	208.2598	615.3034	28.10721
	GH/CHT/SGA	5552.207	816.0145	1084.591	159.4036	5578.291	819.8482	2401.797	352.995
	Pre-eclampsia	8833.204	1684.186	3437.324	655.3786	8607.775	1641.204	1817.837	346.5984
PlGF<12	Normal	0	0	0	0	2850.235	14.15211	1.92E-06	9.56E-09
	GH/CHT/SGA	8373.424	299.3478	6303.676	225.3549	5732.196	204.9246	13675.16	488.8836
	Pre-eclampsia	10374.27	2008.921	7555.648	1463.11	9796.943	1897.124	6127.83	1186.621
